# In Vivo Crystallization of Three-Domain Cry Toxins

**DOI:** 10.3390/toxins9030080

**Published:** 2017-03-09

**Authors:** Rooma Adalat, Faiza Saleem, Neil Crickmore, Shagufta Naz, Abdul Rauf Shakoori

**Affiliations:** 1 Department of Biotechnology, Lahore College for Women University, Lahore 54590, Pakistan; rooma.adalat@gmail.com (R.A.); zoologist1pk@yahoo.com (F.S.); drsnaz31@hotmail.com (S.N.); 2 School of Life Sciences, University of Sussex, Falmer, Brighton BN1 9RH, UK; 3 School of Biological Sciences, University of the Punjab, Quaid-i-Azam Campus, Lahore 54590, Pakistan

**Keywords:** *Bacillus thuringiensis*, C terminal domain, helper protein

## Abstract

*Bacillus thuringiensis* (Bt) is the most successful, environmentally-friendly, and intensively studied microbial insecticide. The major characteristic of Bt is the production of proteinaceous crystals containing toxins with specific activity against many pests including dipteran, lepidopteran, and coleopteran insects, as well as nematodes, protozoa, flukes, and mites. These crystals allow large quantities of the protein toxins to remain stable in the environment until ingested by a susceptible host. It has been previously established that 135 kDa Cry proteins have a crystallization domain at their C-terminal end. In the absence of this domain, Cry proteins often need helper proteins or other factors for crystallization. In this review, we classify the Cry proteins based on their requirements for crystallization.

## 1. Introduction

*Bacillus thuringiensis* (Bt) is a gram-positive, spore-forming bacterium which forms large parasporal crystals during sporulation. These Cry toxin containing crystals have insecticidal activity against many insect orders such as Coleoptera, Lepidoptera, and Diptera, and also against some nematodes, protozoa, and mites [1]. Bt based biopesticides are environmentally-friendly and are widely used for the control of forest and agricultural pests, as well as of vectors of human disease [1,2]. These toxins have also been used as alternatives or supplements to synthetic chemical pesticides [3]. To date more than 700 *cry* genes belonging to 74 classes have been described (http://www.lifesci.sussex.ac.uk/home/Neil_Crickmore/Bt/intro.html)

The high level of Cry protein synthesis is regulated at many levels, including transcriptional, posttranscriptional, and posttranslational, and includes effective promoter function, stable mRNA, co-expression, and assistance by accessory proteins [4,5,6,7,8]. Cry protein synthesis is driven by strong sporulation dependent promoters [4]. The production and accumulation of Cry proteins in the form of crystals during sporulation may help protect the protein from degradation by the proteolytic enzymes that are produced during stationary phase [9]. 

The three-domain Cry (3d-Cry) toxins are globular molecules containing three distinct domains. Domain I is an alpha-helical N terminal domain. Domains II and III are predominantly beta-sheet in nature. One particular feature of the members of the 3d-Cry group is the presence of protoxins with two different lengths of ca. 65 and 135 kDa [6]. The 135 kDa Cry proteins, such as Cry1A, are protoxins in which the actual toxin is at the N-terminus. The C-terminal half has no toxic function, yet is highly conserved among the large set of 135 kDa toxins [10]. It helps in the crystallization of Cry toxin after production [11]. It has been previously established that 135 kDa toxins without their C-terminal half are unable to crystallize within the Bt strain. This so-called crystallization domain comprises 15 to 19 cysteines and contributes to the formation of intermolecular disulfide bonds which stabilize the crystal [12]. In the reducing environment of an insect gut, the cross-links dissociate, releasing protoxin which then undergoes proteolysis to the mature toxin core [13]. Many of the cysteine residues are located on flexible loops in the protoxin domain [10].

The C-terminal extension of the large Cry protoxin has been used as a peptide tag to facilitate the crystallization of recombinant fusion proteins. Hayakawa et al. [14] used a peptide tag derived from the C-terminal half of the Cry4Aa protoxin (amino acids 696–851) to enhance the production and purification of functional TpN protein [14].

3d-Cry proteins of 65 kDa have no C-terminal domain and consist solely of the toxin domain. However, most of these naturally truncated toxins still crystallize readily in Bt. In many cases, other factors are known to be involved, including elements upstream or downstream of the *cry* genes termed “crystallization” [4] or “helper” proteins [15]. 

Here we discuss factors affecting the crystallization of such 3d-Cry proteins. We have split our discussion into four separate sections:

- Separate crystallization domain open reading frames (ORFs)
- Other known crystallization factors
- Toxins with putative crystallization factors
- No known crystallization factors

## 2. Separate Crystallization Domain ORF

Some three-domain Cry toxins have a typical 135 kDa toxin gene that is divided into two separate ORFs. The products of the two ORFs resemble the C-terminal half and the N-terminal half of a 135 kDa toxin. Examples of such Cry proteins are given below, grouped by their known activity.

### 2.1. Mosquitocidal Bt Toxins 

**Figure 1:**
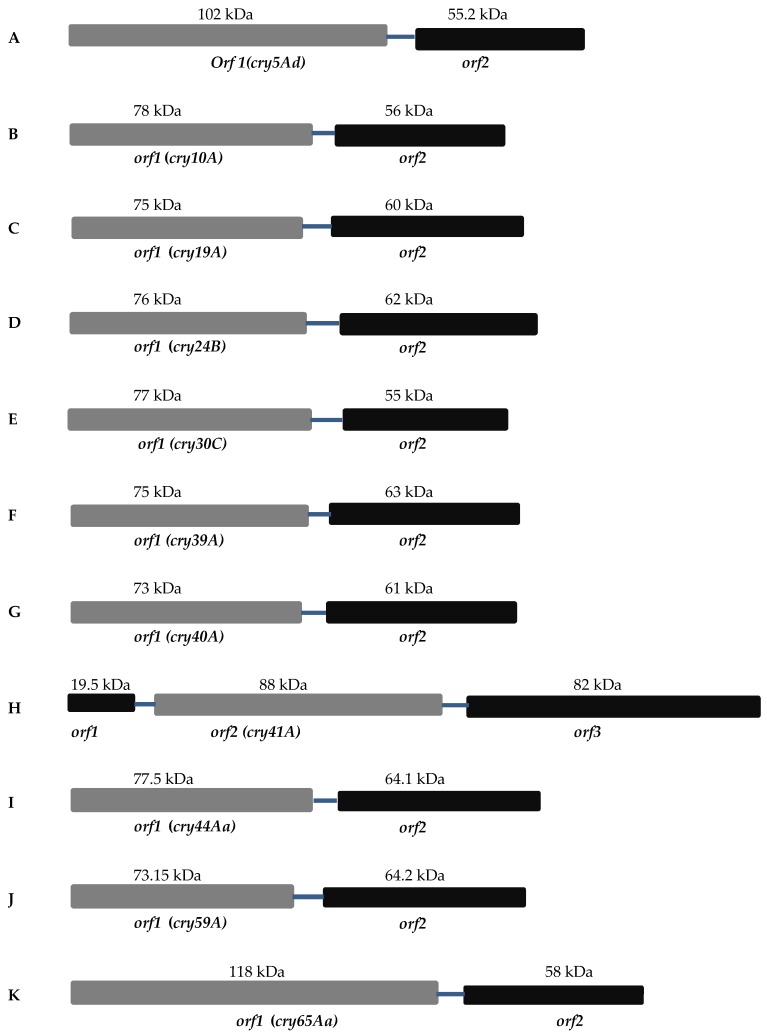
Organization of operons having separate C-terminal domain open reading frames (ORFs): (**A**) *cry5Ad* from *Bacillus thuringiensis* (Bt) strain L366; (**B**) *cry10A* from Bt subsp. *israelensis*; (**C**) *cry19A* from Bt subsp. *jegathesan*; (**D**) *cry24B* from Bt subsp. *sotto*; (**E**) *cry30C* from Bt subsp. *jegathesan*; (**F**) *cry39A* from Bt subsp. *aizawai*; (**G**) *cry40A* from Bt subsp. *aizawai*; (**H**) *cry41A* from Bt strain A146; (**I**) *cry44A* from Bt subsp. *entomocidus*; (**J**) *cry59A* from Bt Bm59-2; (**K**) *cry65Aa* from Bt strain SBT-003.

Figure 1 shows the configuration of various mosquitocidal Bt toxin genes including *cry10A* [16], *cry19A* [17,18], *cry24B* [19], *cry30C* [20], *cry39A* [21], *cry40A* (GenBank accession No. AB074414), *cry44A* [22], and *cry59A* [23]. In each case, the upstream ORF encodes the toxin and the downstream one is the crystallization domain. Barboza-Corona et al. [17] determined the functions of two proteins by expressing different combinations of Cry19A, ORF2, and the N- or C-terminal half of Cry1C in the acrystalliferous Bt strain 4Q7. Their results confirmed that ORF2 of Cry19A assisted in the production and crystallization by functioning like the C-terminal domain of a 135 kDa toxin. 

The length of the intergenic regions is 48 bp, 145 bp, and 114 bp in *cry10A, cry19A,* and *cry30C,* respectively [16,18,20]. There is no homology between these regions and any known transposon or insertion element sequence that could explain the gene separation. Given the high degree of conservation between the ORF2 genes and the C-terminal extensions of the larger toxins, it seems likely that one configuration evolved from the other [24]. It is suggested that the two-gene operon of *cry19A* is the result of mutations that accrued in the extant separating region between *cry19A* and *orf2*. However, factors responsible for this arrangement are still unknown. It may have evolved through the insertion of a DNA fragment into a gene that previously encoded a 135 kDa protein [17,20].

### 2.2. Nematocidal Bt Toxins 

Lenane et al. [25] identified a new member (*cry5Ad*) of the nematocidal *cry5A* family of Bt *cry* protoxin genes. This represents another example of the split gene family described above. The genes encoding Cry5Ad (931 aa) and ORF2-5Ad (502 aa) are organized in an operon and separated by 77 bp in Bt *strain* L366 (Figure 1). Cry5Ad and Orf2-5Ad are distinct from the other members of this family (Cry5Aa-c), which are expressed as single 135–150 kDa δ-endotoxins. It was suggested that the Cry5Ad ORF2, as a major component of the protein crystal, accomplished all the functions ascribed to the C-terminus of the other members of this family [25].

### 2.3. Parasporin Toxins

Parasporins are Bt Cry toxins that have no known insecticidal activity, but are toxic to a variety of human cancer cell lines. Two classes of these are split 3d-Cry toxins.

#### 2.3.1. Cry41A

The Cry41A toxins are 88 kDa in size and when treated with proteinase K release a 64 kDa protein with cytocidal activity against HepG2 (liver hepatocyte) and human cancer cell line HL60 (myeloid leukemia) [26]. Structurally, *cry41Aa/Ab* are three gene operons with three ORFs in the same orientation, as shown in Figure 1. Sequence analysis identified ribosome binding sites associated with all *orf*s. ORF1 of *cry41Aa* encodes 180 amino acid residues (predicted M.W 19.5 kDa), but has no known role in the expression or function of this toxin. ORF2 contains the five conserved blocks found in other insecticidal three domain toxins and encodes an 825aa protein. At the C-terminal end of ORF2 is a 110 amino acid conserved beta-trefoil “ricin” domain containing tandem repeats of QXW/F motif [27]. This is similar to a HA-33 like domain present in the *Clostridium botulinum* type C mammalian neurotoxin that causes botulism disease. [28]. The HA-33 component is responsible for haemagglutination and aggregation of erythrocytes caused by the toxin complex from *Clostridium botulinum*. 

The third gene (*orf3*) encodes a polypeptide with a predicted molecular weight of 82 kDa. It possesses six to eight conserved blocks which show similarity with the C-terminus of the larger 3d-Cry toxins. Cry41Ab shows gene organization similar to Cry41Aa. Krishnan [29] investigated the role of the ORFs and of the ricin domain in crystal and protein production by individually deleting them from the operon. There was apparently no role of ORF1 or the ricin domain, since their deletion did not affect protein expression. Deleting ORF3 resulted in the reduced expression of Cry41Aa/ORF2 and the formation of insoluble inclusions, indicating that ORF3 is required for proper expression [29].

#### 2.3.2. Cry65Aa1

Another interesting example for this type of operon organization is the 118 kDa Cry65Aa1 toxin which reportedly requires two C-termini for crystallization [30]. The N-terminus of Cry65Aa1 resembles a typical three-domain Cry protein. The C-terminal domain is only 36.7 kDa in contrast to the 55–65 kDa domain of the 135 kDa 3d-Cry toxins (Figure 1). Furthermore, there is little sequence similarity between the Cry65Aa C-terminal domain and those of the 3d-Cry toxins. The operon contains a downstream gene encoding an ORF which, like the examples described above, resembles the C-terminal half of the 135 kDa 3d-Cry toxins. This downstream ORF was shown to be required for the efficient expression and crystallization of the Cry65Aa toxin. Peng et al. [30] claimed that the unusual C-terminus of Cry65Aa is also required for crystallization, although this was not clearly demonstrated. Interestingly this report described how a stem-loop structure within the *orf2* gene sequence can aid expression by stabilizing the *cry65Aa* mRNA. 

## 3. Other Known Crystallization Factors 

For a number of other Cry toxins, additional factors that are unrelated to the C-terminus of the 3d-Cry proteins have been shown to be necessary for in vivo crystallization of the toxin. These are discussed below. 

### 3.1. Cry2A Toxins and the ORF2 Repeat Proteins

**Figure 2:**
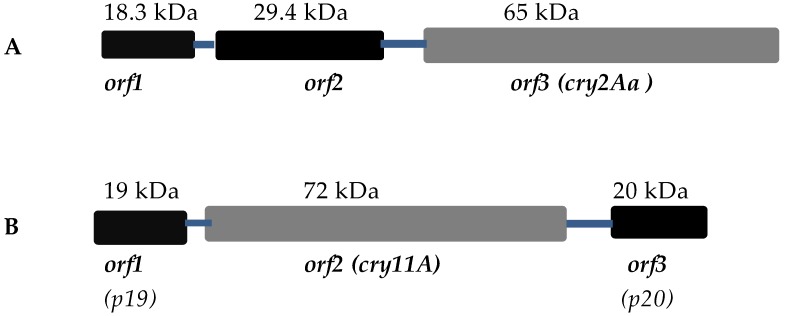
Schematic illustration of Cry toxin operons including potential chaperone genes from Bt subsp. *kurstaki* (**A**); Bt subsp. *israelensis* (**B**).

The genes encoding many of these toxins are also found in the form of operons [31,32,33,34]. In the case of the *cry2Aa* and *cry2Ac* operons, the toxin genes are associated with two other upstream genes, *orf1* and *orf2* (Figure 2). The deletion of *orf1* had no effect on the production of Cry2Aa or Cry2Ac toxins in Bt [32,35]. In contrast, deletion of the *orf2* gene from the *cry2Aa* operon prevented the formation of regular sized crystals even though some toxin was still expressed [35]. It was later shown that Cry2Aa expressed in the absence of ORF2 was distributed randomly in the sporulating cell rather than being concentrated in the cuboidal crystal [36]. The 29 kDa ORF2 protein from the Cry2Aa operon contains a tandem array of eleven repeats of a 15 amino acid motif [31] and it has been speculated that these might form a framework for the crystallization of the Cry2 toxin. Repeat sequences were also identified in the C-terminal region of various other toxins including Cry19Aa, Cry19Ba, Cry20Aa, Cry27Aa, and Cry39/40 [24,36] although the role of these, if any, in crystallization is unknown**.** Although the *cry2Ab* gene is found in many Bt strains, it is not expressed to any significant extent. Crickmore et al. [37] found similarity in the 130 nucleotides upstream of the structural genes between *cry2Ab* and *cry2Aa/Ac* and speculated that *cry2Ab* may once have been part of a similar operon structure. When Cry2Ab was expressed in tandem with ORF2 from the Cry2Aa operon, crystals were then formed [37]. 

It has been reported that DNA fragments are an integral component of some crystals and form protoxin-20 kb DNA complexes [38]. Such a complex might function in crystal formation during the sporulation phase [39,40]. DNA in Cry1 crystals and ORF2 in Cry2Aa crystals could have a similar function, as the highly acidic nature of the Orf2 repeats might mimic the charged phosphate groups on the DNA backbone [36]. However, there is a need to investigate the nature of the association between DNA and Cry protein, as well as the function of DNA in the generation of the protoxin and the stability of the protein [41].

### 3.2. Cry11A and p20

Cry11Aa (formerly known as CryIVD, [42]) is the most active toxin found in the Bt subsp. *israelensis* crystal, which is effective against a range of mosquito larvae [43]. The toxin is encoded by a gene found in a three-gene operon comprising *cry11A* (72 kDa), *p19*, and *p20* [44,45] (Figure 2). SigE controls the transcription of *cry11Aa* [31]. The first ORF (p19) of the *cry11A* operon encodes the p19 polypeptide, which shows significant sequence similarity with the *orf1* gene of the Bt *cry2Aa* and *cry2Ac* operons. The amino acid composition of p19 reveals about 11.7% cysteine residues, like the C-terminal halves of the 135 kDa endotoxins. Dervyn et al. [45] suggest that it might be acting as a chaperone protein and via protein-protein interactions confer a particular lattice structure to allow the co-assembly of the Cry11A inclusions with the other Bt subsp. *israelensis* toxins; however, deletion of the *p19* gene had no detectable effect on Cry11Aa synthesis. Therefore, the role of the 19 kDa protein (*orf1*) remains unclear [45,46]. 

The *orf3* gene of the *cry11A* operon encodes a protein of 20 kDa that functions as a molecular chaperone. When this gene was removed from expression constructs, no Cry11A crystals were observed in Bt. p20 was effective in enhancing the synthesis of Cry11Aa, as it facilitates the formation of larger Cry11Aa crystals in acrystalliferous Bt strains and in recombinant *Escherichia coli* [45,47,48]. The mechanism of Cry11A stabilization by p20 is not known, but it seems to have some functional role in crystal synthesis in Bt [45,49,50]. This protein may be involved in post-translational processing (e.g., it may protect the nascent polypeptide from proteolysis by binding with it and allowing crystal formation) [46]. Moreover, p20 is known to increase the expression levels of other toxins such as Cyt1Aa, Cry1Ab, Cry1Ac, Cry2A, Cry3A, and truncated Cry1C proteins [34,50,51,52,53,54]. 

Dervyn et al. [45] suggested that many of the Cry toxin gene configurations that we see today may have evolved from a common three-gene operon. In some cases, one or both of the non-toxin genes may have been lost, resulting in monocistronic organization. Loss of *orf2* would lead to a situation as seen with *cry10A.* For genes such as *cry2A* [31,32,33] and *cry11A*, all of the three genes are still present [45].

## 4. Toxins with Putative Crystallization Factors

**Figure 3:**
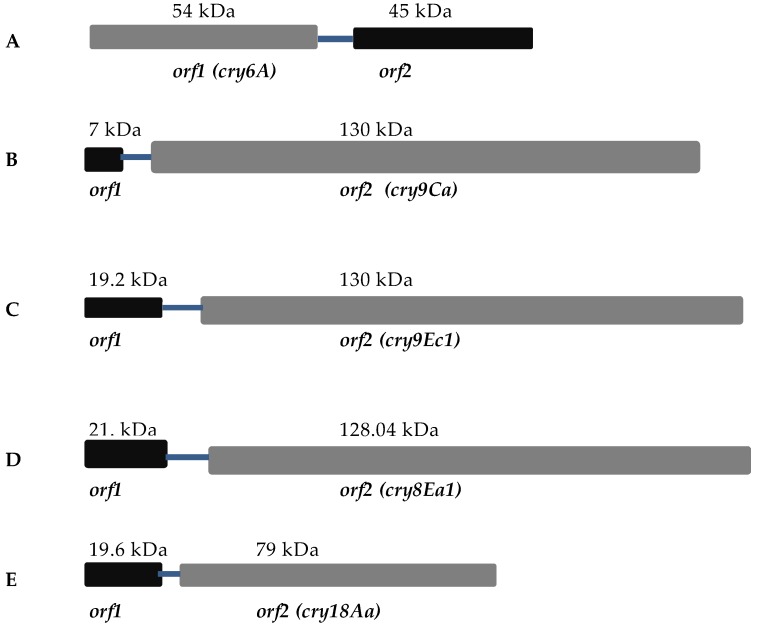
Graphical representation of proteins with putative crystallization factors: (**A**) Cry6A from Bt strain YBT-151; (**B**) Cry9Ca from Bt subsp. *Jegathesan*; (**C**) Cry9Ec from Bt serovar *galleriae*; (**D**) Cry8Ea from Bt strain, Bt185; (**E**) Cry18Aa from *Paenibacillus popilliae*.

There are many Cry toxin genes found in operons (Figure 3) in which no function has been ascribed to the other ORFs present. Some examples of these are described below.

### 4.1. Cry6A

Cry6A crystals, which are toxic against root-knot nematodes, are rice-shaped and produced by Bt strain YBT-151 [55]. The strain inhibits growth, reduces brood size, and decreases mobility and feeding of parasitic nematodes [56]. Yu et al. [57] cloned and characterized the *cry6A* operon containing a structural gene (*orf1*) that encodes 54 kDa toxin having nematicidal activity, a regulatory sequence (stem-loop), and a regulatory gene (*orf2*) encoding 45 kDa (Figure 3)*.* Unlike other Cry proteins, the *orf2* gene negatively regulates *orf1* gene expression and shares no homology with any known sequence. The regulatory sequence acts as a *cis*-factor and results in low level *orf2* expression. High level of *cry6A* expression is due to involvement of two regulatory factors, a stem-loop and *orf2* [57]. It is possible that high levels of expression facilitate crystallization in the absence of any structural role for these ORFs.

### 4.2. Cry9Ca

Cry9Ca is toxic against the important lepidopteran pest, *Ostrinia nubilalis,* as well as secondary pest insects such as armyworms and cutworms. The *cry9Ca* operon consists of two *orfs*, the *cry9Ca* toxin gene and an additional *orf* (Figure 3). The 129.8 kDa toxin is encoded by a second *orf* of the operon. The 7 kDa protein product of *orf1* shows sequence similarity with ORF1 protein of the *cry2Ac* operon. In analogy with the findings of Wu et al. [32], it seems unlikely that this ORF would play an important role in the expression of the Cry9Ca1 crystal [58].

### 4.3. Cry9Ec

The Cry9E*c* toxin consists of 1154 amino acids with high toxicity against *Plutella xylostella* [59]. Upstream from the toxin gene a separate *orf* and a putative promoter region were located, forming a deduced operon (Figure 3). Analysis of the upstream sequence identified a putative promoter region that was homologous to that of the *cry2A* gene recognized by the sporulation-specific Sigma E factor [60]. It is well known that insertion sequence elements are often located in the vicinity of *cry* genes [61]. However, no insertion sequence elements were found adjacent to the *cry9Ec1* gene. The *orf1* gene was identical to various *orfs* in Bt operons [32,58]. The putative 19.2 kDa protein encoded by the *orf1* gene was not observed in parasporal inclusions of the Cry9Ec-expressing strain 92-KU-149-8. Thus, it seems unlikely that ORF1 plays an important role in the production of the Cry9Ec protein [59].

### 4.4. Cry8Ea

The Cry8Ea toxin specifically shows activity against larvae of the Asian cockchafer (*Holotrichia parallela*) [62]. Cry8Ea can be isolated as a 130 kDa protein from spherical inclusions found in Bt strain BT185 [63]. The gene encoding Cry8Ea exists in an operon with an upstream gene *orf1* (Figure 3). In this operon, *orf1* is located 286 bp upstream of *cry8Ea*. An interesting fact is that two promoters initiate transcription of the *cry8Ea* gene. One promoter, Porf1, is present in the upstream region of the *orf1* gene and controlled by the SigE factor. The other, Pcry8E, is found in the intergenic region between *orf1* and *cry8Ea* and is controlled by the SigH factor [31,33,45,64]. This is a case in which *orf1* and *cry8Ea1* form an operon and the *cry* gene can be transcribed either as a bicistronic message or as a monocistronic one [65]. As mentioned above, DNA has been proposed as an important component of crystals which specifically interacts with the protoxin [12,39]. Guo et al. [41] found that DNA and Cry8Ea form a compact complex. DNA is proposed to help in protecting the protein from aggregation and also to enhance the tendency of the toxin to move towards the phospholipid membrane [41]. It has been reported that DNA is degraded by DNases in the midgut of the insect, which is followed by the release of activated toxin and binding with receptors on the apical microvilli of the insect midgut cells [66].

### 4.5. Cry18Aa 

Cry18Aa is a 79 kDa protein found in the sporangia of a strain of *Paenibacillus popilliae* isolated from a diseased larva of the common cockchafer. The gene encoding Cry18Aa is found in a two-gene operon. The first gene encodes a 19.6 kDa ORF1 (Figure 3). The *cry18Aa* gene is located 76 bp downstream of *orf1* [64]. There is no putative promoter between the *orfs*. The *orf1* gene is similar to *orf1* of the *cry2Aa* [31] and *cry2Ac* [32] operons, to *p19* of the *cry11A* [45] operon, and to the first *orf* of the *cry9Ca* [58] operon. When sequence upstream of *orf1* was removed, Bt cells did not produce Cry18A, but the removal of *orf1* itself from the operon had no effect [64].

## 5. No Known Crystallization Factors

There are some 65 kDa-type Cry toxins, such as Cry3A and Cry1I, with no known crystallization factors. These are described below.

### 5.1. Cry3A

Cry3A is an example of a *cry* gene in which high expression levels and crystallization are achieved without the requirement of any known crystallization factor. The Cry3 toxins (i.e., Cry3A, Cry3B, and Cry3C) are active against insects of the order Coleoptera and have been reported from Bt subsp. *morrisoni, galleriae, tenebrionis*, and *tolworthi* [67,68,69,70]. Cry3A proteins are 70 kDa protoxins that are expressed under the control of σ^A^, a sigma factor active during vegetative growth in the predivisional cell [6]. It forms rhomboidal crystals after synthesis [71]. These are toxic to coleopteran insects and also require proteolytic activation. Although there are no known crystallization factors, much is known about the expression of Cry3 toxins. 

#### 5.1.1. Role of Cry3A Domains in Crystallization

The amino acid sequence of Cry3 proteins corresponds to the N-terminal half of the 135 kDa Cry protoxins, in essence making these naturally truncated versions of the latter. As these proteins have no C-terminal crystallization domains characteristic of Cry1 toxins [4], other domains may facilitate Cry3A crystallization. Domain substitution and mutagenesis suggested that the specific structure of a conserved block, which spans the junction between domains I and II, was important for the relative stability of Cry3A and its subsequent crystallization. However, residues of Cry3A other than in helix α7 must also be involved in crystallization, although they remain to be identified [72].

#### 5.1.2. STAB-SD Sequence

Synthesis of *cry3* involves additional transcript stabilization during translation. Studies revealed that the so-called STAB-SD sequence was found downstream of the 5′ end of the major *cry3A* transcript (T-129) and played an important role in stabilizing the Cry3A transcript, assisting net protein production [4,73]. The function of this sequence is to stabilize mRNA by preventing 5′ to 3′ exoribonuclease degradation, and hence is not directly involved in translation initiation [5]. Mathy et al. [74] reported the role of RNase J1 in the exoribonuclease degradation of the −558 transcript of *cry3A* mRNA in the 5′ to 3′ direction. The interaction of 30S ribosomal subunit with STAB-SD sequence blocks the activity of RNase J1 [74].

### 5.2. Cry1I

Tailor et al. [75] reported that CryV, subsequently re-named Cry1Ia [71], is toxic to larvae of both Lepidoptera and Coleoptera. The *cry1Ia* gene cloned from Bt subsp. *kurstaki* DSIR732 produces a protein of 719 amino acids. In Bt strains, this class of toxin gene appeared silent. However in *E. coli*, the gene can be heterologously expressed and produces an 81 kDa protein that is biologically active [76].

Masson et al. [77] observed that the encoded Cry1I protein did not accumulate in crystals in Bt strain HD-133 [78,79]. The Cry1I protein therefore could not be found in the bacterial cell after the T5 sporulation stage [80,81]. This gene was found approximately 500 bp downstream of a *cry1A* gene. No upstream promoter-like sequence could be found [76,81,82]. Nevertheless, identified transcripts showed transcription of *cry1I* mRNA, but the absence of protein product suggested that translation was impaired in some way in Bt [77]. 

## 6. Conclusions 

Further research on *cry* gene organization may give us a better understanding of the structural and functional basis of crystallization factors in Bt. This has significance in biotechnological applications involving the production of these biopesticides. Understanding crystallization in Bt provides better knowledge and important new insights which can result in the expression of heterologous proteins as biologically active crystal inclusions.
